# Supreme Judicial Court

**Published:** 1869-06

**Authors:** 


### THE BOSTON DENTAL COLLEGE.

SUPREME JUDICIAL COURT—Suffolk, ss.—June 19.

Colt, J.—*The Attorney General by Relation vs. The Boston Dental College et al.*—This was an information at the relation of John P. Ordway and others, constituting a minority of the Board of Trustees of the Boston Dental College, to restrain the respondents, the majority of the Board, from conferring the degree of "Doctor of Dental Surgery" upon certain candidates who have been recommended for such degree by the faculty of the College. By the act of incorporation, passed June 3, 1868, the Trustees of the College have authority to confer the degree of "Doctor of Dental Surgery" upon candidates therefor who, upon satisfactory examination by the faculty, have been recommended to the trustees for the degree, provided the candidates shall have devoted three years to the study of Dentistry with a practitioner of Dental Surgery, who shall be approved by the faculty, or shall have been in the practice of Dental Surgery for eight years, *including two full courses of lectures*, the last of which to be pursued in the Boston Dental College.

At the hearing on Saturday, on a motion for an injunction, it appeared that the college was opened for the instruction of students, in the month of September last, and that it has been in operation about ten months, but that during this time the students have attended lectures in the afternoon and evening, so that as alleged by the majority of the trustees they have attended "two full courses of lectures" within the meaning of the act of incorporation.

It was, however, contended on behalf of the complainants, the minority of the trustees, and evidence was offered to show that, according to the usage of all Medical and Dental Colleges, only one "full course" of lectures can be attended by a student during a single academic year, and that accordingly the candidates who have attended lectures but ten months have not attended "two full courses" of lectures within the meaning of the act, that the conferring of the degrees will defeat the purpose and be contrary to the desires of the persons who have contributed money for the support and uses of the college and will not promote the advancement of Dental science and art.

After hearing the evidence the judge ruled that the words of the charter must be construed according to the usage of other Medical and Dental Colleges, and that when so construed the words "two full courses of lectures" mean courses of lectures extending over two academic years.

The injunction was accordingly granted, and it being the purpose of the parties merely to obtain the opinion of the court upon the true construction of the charter the injunction was by consent made perpetual.

A. A. Ranney for the complainants, and B. E. Perry for the defendants.

